# Targeting DNA Methylation Machinery in Pediatric Solid Tumors

**DOI:** 10.3390/cells13141209

**Published:** 2024-07-18

**Authors:** Camilla Cristalli, Katia Scotlandi

**Affiliations:** Laboratory of Experimental Oncology, IRCCS Istituto Ortopedico Rizzoli, Via di Barbiano, 1/10, 40136 Bologna, Italy

**Keywords:** DNA methylation, pediatric solid cancers, DNMT inhibitors

## Abstract

DNA methylation is a key epigenetic regulatory mechanism that plays a critical role in a variety of cellular processes, including the regulation of cell fate during development, maintenance of cell identity, and genome stability. DNA methylation is tightly regulated by enzymatic reactions and its deregulation plays an important role in the development of cancer. Specific DNA methylation alterations have been found in pediatric solid tumors, providing new insights into the development of these tumors. In addition, DNA methylation profiles have greatly contributed to tune the diagnosis of pediatric solid tumors and to define subgroups of patients with different risks of progression, leading to the reduction in unwanted toxicity and the improvement of treatment efficacy. This review highlights the dysregulated DNA methylome in pediatric solid tumors and how this information provides promising targets for epigenetic therapies, particularly inhibitors of DNMT enzymes (DNMTis). Opportunities and limitations are considered, including the ability of DNMTis to induce viral mimicry and immune signaling by tumors. Besides intrinsic action against cancer cells, DNMTis have the potential to sensitize immune-cold tumors to immunotherapies and may represent a remarkable option to improve the treatment of challenging pediatric solid tumors.

## 1. Introduction

The development and progression of several types of cancer have been largely attributed to genomic instability, extensive chromosomal aberrations, and widespread genetic mutations. The classical view is that cells acquire mutations that enable them, with the support of microenvironmental stimuli, to transform into a proliferative, immune-evading, cell-death-resistant state that enables uncontrolled growth and subsequent tumor formation. However, either sequencing data from healthy individuals showing that normal-appearing non-transformed tissues (i.e., normal phenotype) contain millions of cells with multiple oncogenic mutations [1,2], or single cell studies highlighting the clinical relevance of epigenetic intra-tumoral heterogeneity, have forced researchers to embrace the dynamic complexity of tumor development. There is increasing evidence that the non-mutational epigenetic reprogramming of regulatory circuits and networks may contribute to the acquisition of cancer hallmarks during tumor initiation and progression [3].

Epigenetic mechanisms, which include DNA methylation, histone modification, and non-coding RNA regulation, play a crucial role in cancer initiation [4,5] and progression [6,7]. Aberrant DNA methylation patterns, such as the hypermethylation of tumor suppressor gene promoters and global hypomethylation, are common in cancer. Hypermethylation can silence crucial tumor suppressor genes, preventing them from regulating cell growth and apoptosis, while hypomethylation can lead to genomic instability and activation of oncogenes [8,9,10].

Acetylation of histone tails is typically associated with gene activation, while histone methylation can either activate or repress gene expression depending on the specific histone and the location of the modification (e.g., H3K4me3 is associated with gene activation, while H3K27me3 is associated with gene repression). The dysregulation of these modifications can lead to an aberrant expression of genes involved in cell cycle regulation, DNA repair, and apoptosis, contributing to tumorigenesis [11]. The expression of certain miRNAs can be altered in cancer, leading to the dysregulation of target genes that control cell proliferation, differentiation, and apoptosis [12]. LncRNAs can modulate chromatin structure, recruit chromatin-modifying complexes, and interact with miRNAs, further adding to the complexity of gene regulation in cancer [13].

The dynamic nature of the epigenome allows for a heritable yet reversible response to environmental and developmental cues. This plasticity contributes to cancer cell heterogeneity, enabling tumor cells to adapt to various stresses, such as immune surveillance and therapeutic interventions. The resultant epigenetic reprogramming can endow cancer cells with enhanced migratory abilities, resistance to apoptosis, and stem-like properties, facilitating metastasis and treatment resistance during cancer progression [14].

In pediatric cancers, these epigenetic mechanisms are particularly prominent. Pediatric tumors often arise from progenitor cells that have undergone epigenetic alterations, leading to a block in differentiation and the maintenance of a proliferative, stem-like state [3,15,16,17]. The study of these tumors provides valuable insights into the gene regulatory networks driving oncogenesis and highlights potential therapeutic targets.

In this review, we focus on the mechanisms that regulate DNA methylation machinery, outline their relative contribution to pediatric cancers, and discuss their therapeutic potential. The reversible nature of epigenetic alterations, in contrast to the permanence of genetic changes, makes the epigenetic machinery a prime target for the discovery of novel therapeutics. Our review delves into the DNA methylation changes driven by impaired methyltransferase function and histone modifications observed in pediatric solid tumors, offering novel diagnostic and prognostic approaches. We also consider the rapidly evolving knowledge of the impact of DNA methylation changes in tumor immune cell crosstalk as a means to develop new therapeutic approaches.

## 2. Overview of DNA Methylation

Pluripotent stem cells, which have a unique epigenetic plasticity that allows cells to undergo a wide range of lineage commitments, are characterized by a distinct open chromatin configuration that becomes progressively restricted during development [18]. The loss of chromatin accessibility is accompanied by increased DNA methylation and the deposition of specific histone marks that affect chromatin interactions. In particular, in eukaryotes, DNA is organized into nucleoprotein chromatin structures resulting from its wrapping around histone octamers and its decoration with structural and regulatory proteins that lead to the dynamic regulation of histone modifications [19]. DNA methylation and chromatin organization, the templates for epigenetic regulation, are constantly reshaped during early development and differentiation and depend on a complex epigenetic machinery that includes chemical modifications of DNA and histones [20]. This process is mediated by enzymes that can act as ‘writers’ (proteins that add these modifications to target DNA molecules), ‘readers’ (proteins that recognize and interpret these modified codes), and ‘erasers’ (proteins that are responsible for removing these modifications) [21]. The intricate interplay between writers, erasers, and readers orchestrates the dynamic nature of these modifications and regulates transcriptional programs during lineage commitment and stem cells’ self-renewal properties, multilineage differentiation, and proliferative potential [22,23]. Large-scale genetic and proteomic approaches and computational analysis methods have been used to understand how chromatin states are formed, read, and interpreted by nuclear machineries [24,25]. In addition, in the ever-evolving field of epigenetics, RNA modification has also attracted considerable attention and become the subject of extensive investigation. Recent studies suggest that modifications to messenger RNA, such as N6-methyladenosine (m6A), which is the most abundant internal modification found on mRNA, can specifically remove the repressive histone mark H3K9me2 by favoring its demethylation and ultimately promoting gene expression [26]. This indicates a new direct flow of information from RNA to chromatin, adding another layer of regulation to the dynamic epigenome and further highlighting the role of epigenetic signaling in the regulation of gene expression. Several reviews have covered different aspects of the subject [27,28,29].

In this complex scenario, DNA methylation plays a critical role in the regulation of genomic stability, chromatin structure, and gene expression. This process is characterized by the transfer of a methyl group to the fifth carbon (C5) of cytosine within cytosine–guanine (CpG) contexts, resulting in 5-methylcytosine (5-mC). This reaction usually occurs in the context of a CpG site, which is a cytosine nucleotide located near a guanine nucleotide. However, besides the methylation of cytosine at C5, there are other forms of DNA methylation that involve methylation at different nucleotide bases, albeit less common and usually found in specific contexts (e.g., nitrogen N4 of cytosine, N7 of guanine, and N6 of adenine). While these modifications are more commonly studied in prokaryotes and lower eukaryotes, their presence and potential roles in human DNA have also been explored, albeit with less extensive characterization. These modifications has garnered interest due to their potential regulatory roles in gene expression, DNA replication and repair, and DNA metabolism [30,31,32,33]. The methylation status of CpG islands plays a crucial role in gene expression regulation, and its effects can vary depending on the location within the genome. In normal cells, the methylation of promoter CpG islands generally leads to transcriptional silencing. This is achieved by blocking transcription factor binding and recruiting repressive complexes. Methylation can physically obstruct transcription factors from accessing their binding sites while attracting methyl-CpG-binding domain proteins (MBDs) that recruit histone deacetylases (HDACs) and other repressive chromatin remodeling complexes, leading to a more condensed and transcriptionally inactive chromatin [34,35]. Despite most of the CpG islands being located in gene-rich regions within and close to transcription start sites, they are also located in other regions of the genome (e.g., intragenic, 3’UTR, and intergenic). While methylation within gene bodies can affect transcription elongation and alternative splicing, methylation in 3′ UTRs influences mRNA stability and translation efficiency. Intergenic CpG island methylation can regulate enhancer activity, insulator function, and long-range chromatin interactions, thereby exerting control over nearby gene expression [36,37]. Different combinations of histone modifications and DNA methylation create distinct chromatin states (e.g., euchromatin vs. heterochromatin) that regulate gene expression in response to various cues. H3K4me3 is linked to active gene promoters, while its association with H3K27me3 marks bivalent genes poised for activation or repression [20]. In intragenic regions, H3K36me3 is enriched in the exons of actively transcribed genes, promoting transcriptional elongation and alternative splicing [38,39,40]. Histone acetylation within gene bodies enhances transcriptional elongation. In intergenic regions, H3K27ac marks enhancers, activating nearby genes, while H3K9me3 in heterochromatin contributes to gene silencing and chromatin compaction [19,41].

During development, DNA methylation undergoes precise and important modifications [22,34]. In general, DNA methylation is dynamically established and erased at different stages of embryonic development and at different cellular transitions, serving as an epigenetic silencing mechanism for pericentromeric repeats and transposable elements involved in lineage specification, genomic imprinting, and X-chromosome inactivation [20,34,42]. The deposition of repressive epigenetic marks increases after implantation and during embryonic development, while a general decrease in chromatin accessibility is observed during differentiation, limiting the pluripotency potential of stem cells. In fact, while methylation mediates the silencing of pluripotency-associated and gamete-specific genes, a loss of methylation occurs in lineage-specific genes to define cellular identity [43,44]. In normal differentiated tissues, DNA methylation is typically stable and serves to prevent the reactivation of gene expression rather than repression. 

Mechanistically, DNA methylation is carried out by enzymes belonging to the DNA methyltransferase (DNMT) family, which includes DNMT1, DNMT3A, DNMT3B, and DNMT3L [20]. DNMT1 is responsible for maintaining DNA methylation by recognizing hemi-methylated DNA and restoring it to the fully methylated state after DNA replication [45]. The C-terminal catalytic domain of DNMT1 is shared with other DNMTs, but its N-terminal regulatory region is distinct and specifically required for methylation maintenance. It consists of specific domains, including a PCNA binding domain, a nuclear localization signal (NLS), a replication foci targeting sequence (RFTS), and a CXXC domain. The CXXC domain binds to unmethylated CpGs and excludes them from the active site, limiting methylation to hemi-methylated CpGs [46]. The *DNMT1* transcript is continuously expressed in dividing cells and is most abundant during the S phase of the cell cycle due to the recruitment of specific transcription factors [47]. DNMT1 works in close association with the E3 ubiquitin protein ligase UHRF1, and together they are recruited to hemi-methylated sites and regions marked by H3K9 methylation [48]. All of these interactions ensure that DNMT1 activity is stabilized only when engaged in a complex with other heterochromatin-associated proteins and only during DNA replication, providing the fidelity and processivity necessary to replicate a precise methylation pattern. While DNMT1 ensures the faithful propagation of methylation patterns during DNA replication, DNMT3A and DNMT3B, together with DNMT3L (which lacks methyltransferase activity), do not show a preference for hemi-methylated DNA, but are responsible for the establishment of new methylation marks [20]. They mediate histone-directed DNA methylation and share similar domain structures, including a Pro-Trp-Trp-Pro (PWWP) domain that recognizes H3K36me3 in the gene body of actively transcribed genes, and an ATRX-DNMT3L-DNMT3A (ADD) domain that binds to unmethylated H3K4 [49]. All DNMTs use S-adenosylmethionine (SAM) as a methyl group donor and a base flipping mechanism to rotate the target base into the catalytic pocket of the enzyme [50]. 

The process of DNA methylation is reversible and DNA demethylation can be achieved either by the passive replication-dependent dilution of methylated DNA or by active enzymatic demethylation. In active enzymatic demethylation, 5-mC undergoes sequential chemical oxidation by ten–eleven methylcytosine dioxygenase TET proteins (TET1, TET2, and TET3), resulting in the active removal of the label [51]. Alternatively, these modified cytosine species can be cleared during DNA replication if they escape recognition by DNMTs, facilitating passive demethylation [52]. DNMT activity can also be regulated by post-translational modifications. Methylation at two specific lysine residues is the result of the activity of lysine methyltransferase SET7 and lysine demethylase LSD1. When both residues are methylated, DNMT1 is targeted for degradation [50]. In contrast, phosphorylation by the kinase AKT1, which prevents methylation and protects DNMT1 from degradation, links its stability to the phosphoinositide 3-kinase (PI3K)-AKT-mTOR signaling pathway, a key regulator of the cell cycle [50].

## 3. Aberrant DNA Methylation in Cancer

Aberrant epigenetic modifications play a critical role in disease pathogenesis, particularly in cancer. Distinct molecular changes, including variations in DNA methylation, histone-modifying enzymes, and gene expression, are specifically and strongly associated with various cancer types, resulting in altered gene expression patterns that influence crucial biological features of tumor cells, such as growth and invasiveness. Key changes in cancer include abnormal DNA methylation, which in advanced-stage tumors is generally characterized by large hypomethylated intergenic regions compared to adjacent healthy tissues, coupled with hypermethylated specific enhancers/promoters of genes [53]. In general, broad DNA hypomethylation is associated with oncogene activation and chromosomal instability [54], whereas promoter hypermethylation is frequently observed in tumor suppressor genes, leading to their silencing [55]. This contributes to tumor initiation and progression by disabling critical pathways that control cell cycle, apoptosis, and DNA repair. For example, the hypermethylation of the *cyclin dependent kinase inhibitor 2A* (*CDKN2A*) gene promoter, which encodes the tumor suppressor p16, is a common feature in many cancers. The hypermethylation of CpG islands is associated with tumor suppressor and mismatch repair gene silencing [55]. Another example is the epigenetic silencing of the tumor suppressor gene *Von Hippel–Lindau* (*VHL)* via promoter methylation that could predispose one to the development of malignancies, such as clear cell renal cell carcinoma [56]. Furthermore, it has been demonstrated that the gene *O6-methylguanine methyltransferase* (*MGMT*), encoding for a DNA repair enzyme that is responsible for the removal of alkylation adducts on DNA, is frequently hypermethylated in several types of cancer, including glioma and colorectal cancer [57]. Its suppression results in the accumulation of genomic instability and genetic mutations, increasing the occurrence of mutations in genes essential for cellular functions, such as *KRAS* and *TP53* genes [58]. Of note, *MGMT* was the first cancer DNA methylation biomarker to be discovered [59]. Hypermethylation further occurs in bivalent genes, defined by the co-occurrence of activating and repressive histone marks, poised for expression during cell differentiation and subject to Polycomb (PcG)-mediated silencing with the repressive marker H3K27me3 [36,60]. The locking of these genes in a repressed state by DNA hypermethylation prevents proper differentiation, thus contributing to tumorigenesis. Certain regions within gene bodies might retain or gain methylation, which can contribute to oncogene activation. Hypermethylation within oncogene bodies may enhance their expression by stabilizing transcription elongation [61,62]. Mutations in *isocitrate dehydrogenases* (*IDH1* and *IDH2*) lead to the production of 2-hydroxyglutarate (2-HG), an oncometabolite that inhibits the TET2 enzyme’s ability to demethylate DNA. This inhibition results in extensive DNA hypermethylation, silencing important regulatory genes and promoting cancer development and progression [63]. 

The relationship between DNA methylation and gene expression is complex, with DNA hypomethylation representing a necessary but not sufficient condition for determining gene expression. This is because several functional elements can contribute to maintaining the efficiency of transcription [64,65]. Indeed, only a small percentage of DNA hypomethylated regions gain functionality and chromatin accessibility following treatment with Decitabine (5-aza-2′-deoxycytidine), a cytidine analog that inhibits DNMTs through covalent binding [66]. Cancer cell treatment with this drug was shown not only to reactivate genes, but also decrease the overexpression of genes controlled by oncogenes, such as the oncogene c-MYC [62]. Downregulation is caused by the DNA demethylation of gene bodies, and the restoration of their high expression levels requires rapid re-methylation. The authors reported that the rate of re-methylation was strongly influenced by DNMT3B but not by DNMT1, consistent with DNMT1 functioning mainly as a maintenance enzyme. DNA methylation inhibitors may thus lead to the normalization of gene overexpression induced during carcinogenesis and have the potential to redirect cancer cells to a more benign condition. This may be particularly relevant for pediatric cancers, which are often locked into an undifferentiated state due to aberrant DNA methylation at specific chromatin regions involved in stemness or differentiation. However, caution is necessary and understanding the implications of general DNA demethylation in tumors is essential to elucidate the molecular mechanisms underlying cancer progression and identify potential therapeutic targets. In fact, generalized DNA demethylation in tumors can have a range of consequences. It can reactivate tumor suppressor genes and inhibit cancer growth, but it can also activate pro-metastatic genes, potentially increasing metastasis, or it can induce chromosomal instability, leading to genomic alterations that contribute to cancer progression. Moreover, DNA demethylation can reactivate tumor-associated antigens and immunomodulatory pathway genes, which may affect the immune response against cancer [67,68], providing a new area of active investigation. With respect to pediatric solid tumors, the study of DNA methylation machinery has proven useful in diagnosis, prognosis, and therapy. Distinctive genetic profiles of 5-mC were found to specifically categorize nervous system tumors and sarcomas [69,70], opening new perspectives for a group of tumors with still unmet clinical needs. 

## 4. Pediatric Nervous System Tumors

Nervous system tumors represent nearly 30% of all pediatric cancers [71] and include gliomas, medulloblastomas (MBs), and neuroblastomas (NBs). Although there are clear pathological differences among these cancer types, they all originate from stem or progenitor cells whose cell fate is critically regulated by epigenetic mechanisms [72,73]. These mechanisms are crucial not only for determining cell fate, but also for the development and progression of these tumors.

### 4.1. Glioma

Gliomas represent the most common type of pediatric central nervous system (CNS) tumors and originate from glial cells, most likely neuroglial stem or progenitor cells (astrocytes, ependymal cells, and oligodendrocytes) [71,74,75,76]. Historically classified based on its histopathological features, glioma classification now includes molecular markers and DNA methylation profiles. Grades I and II are considered low-grade gliomas (LGGs), while grades III and IV are high-grade gliomas (HGGs), with glioblastomas (GBMs) being the most aggressive [77]. The latest details on diagnosis and clinical management are reviewed elsewhere [78]. Recent findings have highlighted significant differences between adult and pediatric gliomas, which were recognized in the 2021 WHO CNS tumor classification. Adult gliomas commonly have *IDH* mutations, while pediatric gliomas frequently exhibit histone 3 (H3) mutations [79,80]. *IDH* mutations were first discovered in rare GBMs of younger adults [81] and represent the most frequent alteration in LGGs, where they represent a principal mechanism of enhanced histone and DNA methylation referred to as the glioma CpG island methylator phenotype (g-CIMP) [63]. Mutated IDH enzymes exhibit neomorphic activity, producing 2-HG instead of α-ketoglutarate (α-KG). This accumulation of 2-HG disrupts the Krebs cycle, alters cellular metabolism, and induces epigenetic reprogramming [82]. 2-HG structurally resembles α-KG, blocks the activity of TET proteins, and leads to DNA hypermethylation. Additionally, 2-HG inhibits histone demethylases, resulting in increased histone methylation, particularly H3K4me3, H3K9me3, and H3K27me3. The accumulation of these chromatin marks results in impaired cellular differentiation [83]. Even in IDH wild-type tumors, DNA methylation profiling is useful for classification as it can discriminate between subtypes and can predict somatic genomic alterations (i.e., *Telomerase Reverse Transcriptase*, *TERT* activation, *ATRX Chromatin Remodeler*, *ATRX* inactivation, and receptor tyrosine kinases amplifications) particularly relevant in HGGs [69,84]. 

One of the most extensively investigated prognostic biomarkers in neuro-oncology is the DNA methylation status of the *MGMT* gene promoter [85]. MGMT is a repair protein that counteracts DNA damage induced by alkylating agents, such as temozolomide (TMZ), which is the standard chemotherapy used for GBM [86]. The DNA methylation pattern of the *MGMT* gene promoter has been identified as a prognostic marker for GBM patients treated with TMZ. Patients with silenced *MGMT* gene expression due to high promoter methylation tend to have better outcomes compared to those with unmethylated promoters [87]. However, MGMT methylation status has been described to vary within different regions of tumors. This means that treatment decisions could be influenced by which part of the tumor is sampled [88,89]. Unfortunately, in pediatric HGGs, despite the frequent occurrence of MGMT promoter methylation, the prognosis remains poor [56]. 

HGGs account for 10% of pediatric CNS tumors and in approximately 50% of cases are defined by mutations in the histone 3 variant H3.3 that alters key sites of regulatory methylation. Diffuse midline glioma H3 K27-altered presents a substitution of lysine 27 with methionine (K27M) and the diffuse hemispheric glioma H3 G34 mutant presents a substitution in glycine 34 with arginine (G34R) [80]. This leads to the inhibition of the PcG EZH2 enzyme, resulting in a global reduction in the repressive histone mark H3K27me3. This reduction is associated with altered DNA methylation [90,91]. G34R mutation is linked to a hypomethylation phenotype, especially at the ends of chromosomes. In contrast, the K27M mutation has a distinct methylation pattern, different from both G34R and the g-CIMP phenotype associated with *IDH1* mutations [79]. Recent reports suggest that G34R mutations have similar effects to the G34W mutation, which is prevalent in bone tumors. These mutations have characteristic methylomic effects, regardless of the tumor tissue of origin, and may affect chromosome instability [92]. 

DNA methylation is widely regarded as a primary driver of these tumors and studies are directed to exploit this epigenetic mark as a potential therapeutic target. DNMTis, such as decitabine and 5-azacytidine, have demonstrated the ability to suppress IDH-mutated glioma cell proliferation in preclinical studies. Treatment results in the reversal of DNA methylation marks, loss of stem-like properties, and re-expression of PcG-regulated genes associated with differentiation [93,94]. DNA methylation machinery targeting has been demonstrated to influence glioma cells’ malignant phenotype by various mechanisms involving the inhibition of cell growth [95,96,97], proliferation [98], migration [99], and invasiveness [100], with potential therapeutic implications.

**Figure 1:**
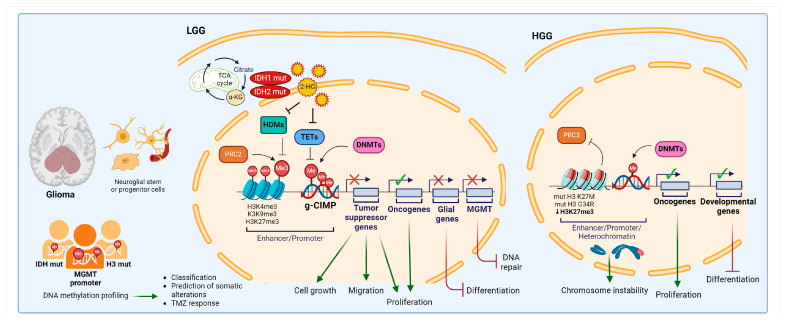
DNA methylation machinery in gliomas. Gliomas, originating from brain glial cells, are classified as low grade (LGG) or high grade (HGG). DNA methylation profiling aids in diagnosis and predicting TMZ therapy response. In LGGs, IDH1/2 mutations produce 2-HG, causing epigenetic reprogramming and increased methylation (g-CIMP phenotype), leading to gene repression and oncogene activation, thus increasing proliferation and blocking differentiation. *MGMT* promoter methylation is a key prognostic biomarker for better TMZ outcomes. Pediatric HGGs often have H3 mutations (K27M, G34R) that alter methylation, causing chromosomal instability and oncogene activation, resulting in increased proliferation and blocked differentiation. LGG, low-grade glioma; HGG, high-grade glioma; IDH, isocitrate dehydrogenase; TCA cycle, tricarboxylic acid cycle; α-KG, alpha-ketoglutarate; 2-HG, 2-hydroxyglutarate; HDMs, histone demethylases, TET, Tet methylcytosine dioxygenase; PRC2, Polycomb repressive complex 2; DNMTs, DNA methyltransferases; g-CIMP, glioma CpG island methylator phenotype; *MGMT*, *O-6-Methylguanine-DNA Methyltransferase*; TMZ, Temozolomide; H3K4me3, histone H3 lysine 4 trimethylation; H3K9me3, histone H3 lysine 9 trimethylation; H3K27me3, histone H3 lysine 27 trimethylation; H3 K27M, histone H3 substitution of lysine 27 with methionine; H3 G34R histone H3 substitution of glycine with arginine.

Figure 1 shows the epigenetic alterations related to DNA methylation described in gliomas.

### 4.2. Medulloblastomas and Embryonal CNS Tumors

MB is the most frequent embryonal brain tumor (1 per 100,000 births) and originates in the cerebellum from neuronal stem or progenitor cells [101]. It is clinically and biologically heterogeneous. The subtypes of MBs are currently characterized by molecular and clinical features [102] and are designated as: group 1 WNT-MB and group 2 Sonic Hedgehog (SHH)-MB, which are characterized by the constitutive active signaling of WNT and SHH, respectively; and group 3 and group 4, which are characterized by a preponderance of somatic copy number aberrations (SCNAs) and structural variants (SVs) [103]. SHH MB was the first MB subgroup to be recognized [104]. Over the years, MB has been extensively sequenced by distinct -omics (that is, genomic, epigenomic, transcriptomic, and proteomic) [105] and, as a result, the molecular classification of MB has evolved beyond the four consensus subgroups to include additional subdivisions (also referred to as ‘subtypes’). Particularly, the definitions and recognition of group 3 and group 4 MBs have continued to evolve during years and recently the WHO [77] formally acknowledge their definition as mostly discrete molecular entities. Analysis of the genomes of group 3 and group 4 MB samples identified c-MYC copy number amplifications mainly restricted to a subtype of group 3 tumors associated with the most aggressive forms of MBs [106]. In group 4, it is reported the frequent activation of PRMD6, a transcriptional repressor, mediates gene silencing through intrinsic histone H4 lysine 20 (H4K20) methyltransferase activity, in concert with known chromatin-associated repressive complexes [107]. More recently, spatial and single-cell genomic approaches also have been applied, delving into the intra-tumoral heterogeneity, cellular composition, and developmental origins of MB at a single-cell resolution [108,109], and new methods for robust and accurate assignments of patients into the relevant subtypes have become mainstream. DNA methylation arrays, which can measure hundreds of thousands of methylation sites across the genome, have emerged as the platform of choice for MB classification. Owing to the relative stability of DNA, methylation arrays and derivative-targeted DNA methylation-based assays allow for the routine analysis of clinical samples in a diagnostic setting as well as the profiling of archival tumor material, with limited technical variation between institutions [69,110,111]. Thus, DNA methylation profiling is now the gold standard for identifying MB subgroups and determining their substructure, surpassing gene expression analysis [112,113,114]. Interestingly, paired DNA methylation and transcriptome analyses of patient samples with MB revealed that the classical notion of gene silencing by promoter hypermethylation is not a prominent feature. Instead, MB shows prevalent regions of hypomethylation that correlate with increased gene expression, extending tens of kilobases downstream of transcription start sites, or patterns of differential DNA methylation in the region of novel exons and transcription start sites that generate alternative transcripts whose expression is particularly subgroup specific [115].

The accurate genetic characterization and classification of MB have clinical and biological implications. Clinically, although cure rates are reasonably good, in the range of 70–75% for all patients with MB, the separation of MBs along molecular pathways into subgroups, and more recently into subtypes within subgroups, has led to the possibility of modulating therapies according to risk, allowing the consideration of reducing therapy for the lower-risk WNT subgroup, while maintaining and optimizing intensive therapy for the high-risk patients within group 3 MBs. Biological mechanistic studies related to molecular subgroup status are now underway, leading to preclinical results and models that more accurately recapitulate human disease. For example, the WNT and SHH subgroups, which represent approximately 10% and 30%, respectively, of all patients with MB, are associated with the activation of the WNT and SHH signaling pathways. The activation of the SHH signaling pathway in MB is associated with the overexpression of DNMT1. This leads to the hypermethylation of tumor suppressor gene promoters, such as *hypermethylated in cancer transcriptional repressor 1* (*HIC1*), *RAS association domain family member 1* (*RASSF1A*), and caspase 8 *(CASP8*), which results in tumor proliferation [116]. Both nucleoside and non-nucleoside DNMTis have been demonstrated to reduce metabolic activity and induce differentiation in MB cells and reactivate silenced tumor suppressor genes [116,117,118,119,120,121]. Combination studies with other drugs, such as chemotherapeutics or HDAC inhibitors, demonstrate synergistic effects [118]. An integrated functional genomics approach has been recently used to identify epigenetic regulators of SHH signaling. The approach identified a novel complex composed of UHRF1, DNMT1, and GLI proteins. Based on this, a DNMT1 inhibitor was shown to be able to halt the progression of MB in an orthotopic SHH-MB mouse model [122].

Besides MB, embryonal tumors with multilayered rosettes (ETMRs) represent another group of CNS embryonal tumors that arise in young children [123] and have a still dismal prognosis, (i.e., the 5-year overall survival rate is less than 10%) despite the use of intensive multimodal therapy [124]. From a genetic point of view, these tumors are characterized by the amplification of the microRNA cluster C19MC on chromosome 19q and by highly distinctive DNA methylation patterns [125,126]. Integrated genetic and epigenetic analysis exhibits a common transcriptional and epigenomic signature with enrichment in pluripotency and early neural differentiation genes [123]. RNA-sequencing studies have revealed recurrent gene fusions of *C19MC* with *TTYH1*, a developmentally restricted chloride channel gene leading to the high expression of specific C19MC miRNAs that in turn induce the expression of a neural-specific DNMT3B isoform [123]. DNMTs and cooperating epigenomic modifiers are potential therapeutic targets for these fatal tumors. In preclinical studies, treatment with DNMTis has been found to inhibit the growth of ETMR cell lines by promoting cell death and differentiation [126,127,128].

**Figure 2:**
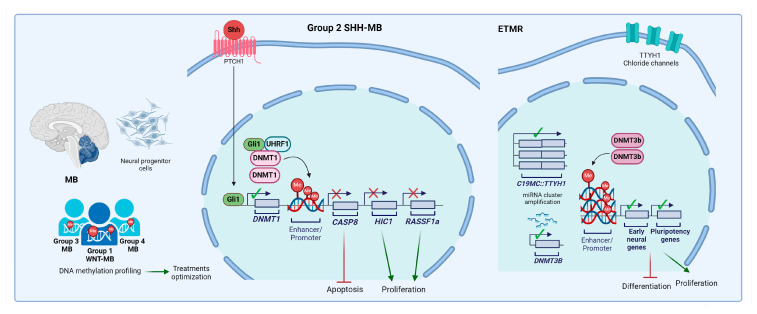
DNA methylation machinery in medulloblastomas. Medulloblastoma (MB), the most common embryonal brain tumor, originates from cerebellar neuronal stem or progenitor cells. MB subtypes (WNT-MB, SHH-MB, group 3, and group 4) exhibit distinct molecular features. DNA methylation profiling is crucial for precise classification and treatment selection. Activation of the SHH pathway in SHH-MB upregulates DNMT1, causing the hypermethylation of tumor suppressor gene promoters inducing proliferation. UHRF1, DNMT1, and GLI proteins have been identified as epigenetic regulators of SHH signaling affecting DNA methylation. ETMRs, another group of CNS embryonal tumors, are characterized by *C19MC* amplification and distinct DNA methylation patterns. Recurrent gene fusions between *C19MC* and *TTYH1*, a developmental-restricted chloride channel, lead to increased expression of specific C19MC miRNAs, which in turn induce the neural-specific DNMT3B isoform. The result is the induction of expression of pluripotency and early neural differentiation genes that block differentiation and promote proliferation. SHH-MB, Sonic Hedgehog signaling MB; Shh, SHH ligand; PTCH1, patched homolog 1 (receptor for the secreted hedgehog ligand); Gli1, GLI family zinc finger 1; UHRF1, ubiquitin-like with PHD and ring finger domain 1; DNMT1, DNA methyltransferase 1; WNT, wingless-type family member signaling; *CASP8*, *caspase 8*; *HIC1*, *hypermethylated in cancer transcriptional repressor 1*; *RASSF1A*, *Ras association domain family member 1*; ETMRs, embryonal tumors with multilayered rosettes; TTYH1, Tweety family member 1 chloride ion channel; *C19MC*, *chromosome 19 miRNA cluster*; *DNMT3B*, *DNA methyltransferase 3B*.

Figure 2 outlines the main alterations involving DNA methylation machinery in MB and EMTR.

### 4.3. Neuroblastoma

NB is a tumor of the peripheral nervous system and is the most common type of extracranial solid cancer in children, with distinctive features such as early onset, frequent metastases at diagnosis, and a notable propensity for spontaneous regression during infancy [129]. It occurs in the developing sympathetic nervous system, including ganglia and the adrenal gland, and is thought to originate from neural crest-derived progenitors, ranging from mesenchymal to more committed adrenal cells [130,131,132]. Features known to be associated with poor prognosis include age greater than 18 months, recurrence of genetic alterations, and chromosomal defects, such as deleterious *MYCN* amplification and chromosome 11q deletion [133,134]. Treatment for NB often involves intensive multidisciplinary therapy, including surgery, chemotherapy, radiotherapy, and stem cell transplantation. However, despite these efforts, survival rates for high-risk cases remain disappointingly low [135]. For further details on NB treatments and current advances please refer to [136].

Comprehensive genomic analysis of a large number of patients has shown that NB cases cannot be explained by a single mutation [137]. In addition, only a minor proportion of patients respond to targeted therapies. Otherwise, epigenetic alterations have been demonstrated to significantly influence tumor development and aggressiveness. Initial studies identified several hypermethylated genes (e.g., *homeobox protein Hox-A9, HOXA9*; *RASSF1A* and *CASP8*), which are associated with tumor suppression, differentiation, and apoptosis, and defined a CIMP methylator phenotype marked by the extensive methylation of multiple CpG islands linked to poor prognosis [138,139,140]. Genome-wide analyses of DNA methylation revealed both established and novel DNA methylated regions, such as telomeric silencing and identified subgroups in NB, correlating with specific genetic and clinical features, such as age, stage, *MYCN* amplification, and immune infiltration [141,142,143,144]. Additionally, further global approaches of methylation-based clustering demonstrated the ability to assess or predict the risk and prognosis of patients and continued to identify novel subgroups of NB, such as high- and low-risk infant patients [145] and an ultra-high-risk tumor group with 11q loss of heterozygosity [146]. Integrative analysis of methylomes, transcriptomes, and histone modification further revealed enhancer methylation as a mechanism for high-risk-associated transcriptional dysregulation and provided evidence that PRC2 activity and DNA methylation co-operate to block differentiation programs [142]. Genetic alterations, such as gains or amplifications of chromosome 7 or 7q, contribute to elevated levels of EZH2, a component of PRC2, in undifferentiated NB with a poor prognosis [147,148]. Additionally, LSD1 was overexpressed in poorly differentiated NB and it preferentially demethylated H3K4me3, thus favoring PRC2-associated H3K27me3 silencing at bivalent loci and the maintenance of stemness [149]. A recent study of the 5-hydroxymethylcytosine (5-hmC) profile in circulating cell-free DNA and tumor biopsies identified increased 5-hmC deposition on PRC2 target bivalent genes. This resulted in significantly and independently associated worse clinical outcomes in patients with high-risk NB [150].

**Figure 3:**
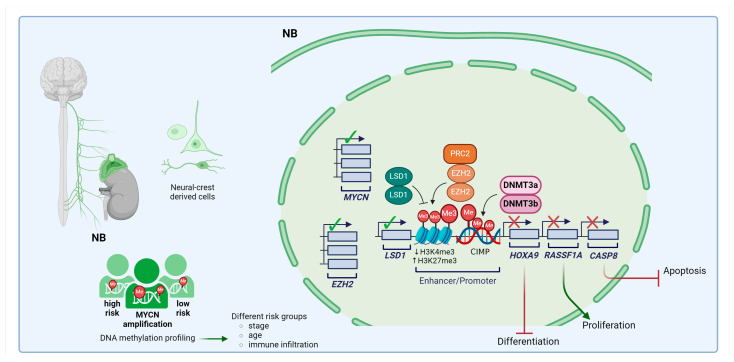
DNA methylation machinery in neuroblastomas. Neuroblastoma (NB) arises in the peripheral sympathetic nervous system, including ganglia and the adrenal gland, from neural crest-derived cells. DNA methylation profiling identified a CIMP phenotype and different NB risk groups associated with poor prognosis, correlated with age, stage, *MYCN* amplification, and immune infiltration. Hypermethylated and repressed genes in NB are linked to tumor suppression, differentiation, and apoptosis (e.g., *HOXA9*, *RASSF1A*, and *CASP8*). High levels of DNMT3A/B correlate with high-risk NB. Overexpression of histone modifiers LSD1 and EZH2 (PRC2 component) in poorly differentiated NB affects H3K4 and H3K27 marks at differentiation genes. PRC2 activity and DNA methylation at enhancers cooperate to block differentiation, contributing to transcriptional dysregulation in high-risk NB. CIMP, CpG island methylator phenotype; *HOXA9*, *Homeobox protein Hox-A9*; *RASSF1A*, *RAS association domain family member 1*; *CASP8*, *caspase 8*; DNMT3A, DNA methyltransferase 3A; DNMT3B, DNA methyltransferase 3B; *EZH2*, *enhancer of Zeste 2 Polycomb repressive complex 2 subunit*; *LSD1*, *lysine demethylase 1*; H3K4me3, histone H3 lysine 4 trimethylation; H3K27me3, histone H3 lysine 27 trimethylation.

All of these findings support the potential of exploring epigenetic interventions to expand the therapeutic options for high-risk NB in an effort to improve patient survival. Preclinical studies investigated the role of DNMTs and their inhibition of NB and found that they influence tumor behavior and response to treatment. In particular, higher levels of DNMT3A/B associates with high-risk NB; conversely, a truncated form, DNMT3B7, correlates with better clinical outcomes [151]. DNMT3B7 overexpression in mice inhibited tumor growth by reducing proliferation, increasing apoptosis, and lowering vascularity. Furthermore, DNMT3B7 enhanced the expression of retinoic acid pathway genes, promoting NB differentiation during all-trans retinoic acid (ATRA) treatment [151]. Retinoic acid-based therapeutics offer significant clinical benefits to NB patients and have been shown to stop disease progression by inducing differentiation through reprogramming adrenergic core regulatory circuits [152]. DNMTis alone or combined with a synthetic retinoid has been shown to improve the efficacy of NB reprogramming, resulting in the rapid downregulation of *MYCN* expression, even in the context of high levels of *MYCN* gene amplification, together with the induction of cell differentiation and proliferative arrest in vitro and in vivo [121,153,154,155,156]. miRNAs also contribute to epigenetic modifications essential for NB differentiation induced by ATRA. They regulate the expression of DNMT1 and DNMT3B, further influencing the epigenetic landscape of NB cells [157,158]. The retinoids’ ability to reactivate differentiation indicates that, despite the genetic alterations observed in high-risk tumors, the regulatory signaling pathways that control growth and differentiation remain functional but are subject to epigenetic repression. Figure 3 illustrates the main alterations in DNA methylation machinery in NB.

## 5. Sarcomas

The principle of methylation-based tumor profiling that has been developed for the comprehensive brain tumor classifier has been recently extended to small, blue, round-cell sarcomas, a subgroup of tumors still evading a definite histological diagnosis [70,159,160,161], demonstrating the potential of the DNA methylation-based sarcoma classification for research and future diagnostic applications. Sarcomas are a heterogeneous group of tumors, including a collection of more than 60 malignancies with diverse biological, genetical, and clinical characteristics [162]. The genetics of sarcomas are highly variable. Some histotypes have nearly diploid karyotypes and are driven by chromosomal translocations, leading to the formation of fusion oncogenes, such as Ewing sarcoma (EwS) [163]. In contrast, other sarcomas, such as osteosarcoma (OS), have complex genomic alterations and are driven by copy number alterations or by mutations [164,165]. Besides genetic alterations, increasing evidence showed that epigenetic deregulation may also play an essential role in the pathogenesis of these tumors. Quite a number of different types of dysregulations have been described. For example, a group of chondrosarcomas are driven by *IDH1/2* mutations, which alter DNA hydroxymethylation and histone demethylation, while giant cell tumors of bone and chondroblastoma are driven by somatic mutations in genes encoding histones, named ‘oncohistones’ [166,167]. In addition, alterations in a chromatin writing complex, or genetic alterations disrupting chromatin remodeling complexes, have been described in malignant peripheral nerve sheath tumors (MPNSTs), synovial sarcoma, malignant rhabdoid tumors, and epithelioid sarcomas [168]. Here, we critically summarize the alterations leading to aberrant DNA methylation patterns in the three most common pediatric sarcomas of bone and soft tissues, i.e., OS, EwS, and rhabdomyosarcoma (RMS).

### 5.1. Osteosarcoma

OS is the most common primary malignant tumor of bone, commonly arising in adolescents and young adults. Whole-exome sequencing or targeted sequencing of the DNA of 1244 patients with OS from 10 participating international centers or studies revealed that approximately one-fourth of patients with OS, unselected for family history, had a highly penetrant germline mutation in genes not previously linked to the tumor (e.g., *CDKN2A*; *Menin 1*, *MEN1*; *VHL*; *Protection Of Telomeres 1, POT1*; *APC*; *MutS Homolog 2*, *MSH2* and *ATRX*) and in the Li-Fraumeni syndrome-associated gene, *TP53* [169]. However, the causes leading to disease onset are still unknown. OS is a genomically complex disease characterized by few recurrent single-nucleotide mutations but frequent, widespread large-scale alterations, which lead to heterogeneous genomic and transcriptomic landscapes [170]. Such heterogeneity has complicated the identification of the key oncogenic drivers underlying disease emergence or progression. However, the inactivation of tumor suppressor genes, such as *TP53*, *RB*, and *ATRX*, and hyperactivation of some proto-oncogenes, including *MYC* and *MDM2*, are frequently observed and recent multi-omics data have led to OS classification into different subtypes with distinct molecular features and clinical prognoses [171,172,173]. 

Despite the fact that OS is considered to be a SCNA-driven tumor, there is a growing interest in profiling DNA methylation as a means of defining tumor-specific methylation patterns with diagnostic and prognostic relevance. OS methylation profiles are distinct from those observed in colorectal cancers and glioma CIMP, and disease-associated methylation signatures can distinguish OS from Ewing and synovial sarcomas [174,175]. Methylation of a single gene or locus has been shown to affect disease progression [176,177]. However, more recent genome-wide studies revealed that primary OS methylation patterns are strongly associated with patient outcomes and that broader methylation correlates better with tumor response to treatments rather than single specific genes [175,178]. In particular, tumors with relatively low methylation levels were found to respond better to chemotherapy than those with high methylation levels [175]. Hypermethylation may protect against DNA damage induced by chemotherapeutic agents, such as doxorubicin or cisplatin. Alternatively, it can even physically disrupt their mechanisms of action by limiting chromatin accessibility. Conversely, genomic hypomethylation is known to cause genomic instability and to increase tumor immunogenicity. This is particularly noteworthy in light of recent immunogenomic findings indicating that OSA tumors with optimal chemoresponse outcomes exhibit fewer stable genomes and higher activation of the immune response compared to those with a suboptimal response [179]. Thus, the assessment of genomic methylation may represent a strategy for stratifying patients for the application of alternative therapies. Of note, a new classifier based on DNA methylation patterns that detects BRCAness in OS samples with high accuracy was recently identified [180]. This may provide a basis for clinical decision making regarding the administration of PARPi in patients with this tumor.

**Figure 4:**
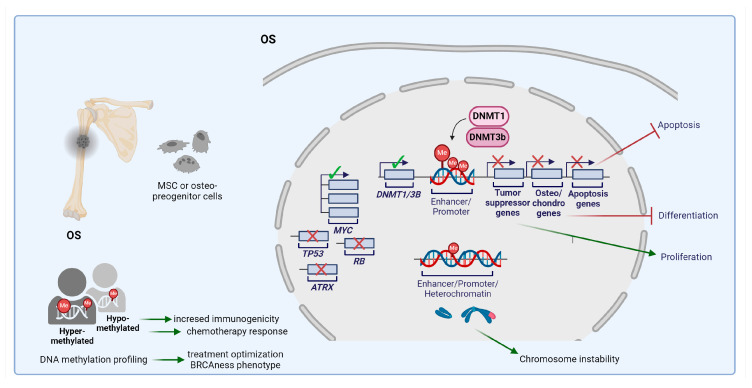
DNA methylation machinery in osteosarcomas. Osteosarcoma (OS), the most common bone tumor in children and adolescents, arises in bone from mesenchymal stem or osteoprogenitor cells. OS exhibits heterogeneous genomic and transcriptomic landscapes with the inactivation of tumor suppressor genes (e.g., *TP53*, *RB*, and *ATRX*) and activation of proto-oncogenes (e.g., *MYC*). DNA methylation patterns are distinctive of OS and correlate with patient outcomes and chemotherapy response. Hypomethylation correlates with a better response to treatment and increased tumor immunogenicity. A new classifier based on DNA methylation patterns detects BRCAness in OS. Overexpression of DNMT1 and DNMT3B was frequently observed in OS. DNA hypermethylation affects several tumor suppressor, osteo/chondrogenesis, and apoptosis genes, while hypomethylation induces chromosomal instability, leading to increased proliferation, reduced differentiation, and decreased cell death. MSCs, mesenchymal stem cells; CIMP, CpG island methylator phenotype; DNMT1, DNA methyltransferase 1; DNMT3B, DNA methyltransferase 3B; BRCAness, phenotypic copy of germline BRCA mutations; *ATRX*, *Alpha Thalassemia/Mental Retardation Syndrome X-Linked chromatin remodeler*; *RB*, *retinoblastoma*.

Preclinical studies have demonstrated the efficacy of DNMTis in the treatment of OS. In comparison to normal cells, the overexpression of DNMT1, together with copy-number gains and related overexpression of DNMT3B, was frequently observed in OS [181,182]. DNMTis, including the FDA-approved decitabine and the novel non-nucleoside DNMTis, MC3343 that specifically inhibits DNMT1 and DNMT3A [183], were found to inhibit OS cell growth, to induce osteoblast differentiation, to reduce metastasis, and to synergize with both doxorubicin and cisplatin in OS cell lines [176,177,184,185,186,187]. In their search for a mechanistic explanation of drug efficacy, Asano et al. discovered that multiple tumor-suppressor and osteo/chondrogenesis-related genes were reactivated by decitabine treatment of OS cells [176]. Similarly, combined DNMTi and HDACi treatment of multidrug-resistant OS cells in vitro revealed the re-expression of epigenetically silenced genes involved in p53-independent apoptotic signaling and osteoblast differentiation pathways [188,189], while DNMTi MC3343 efficiently induced the specific re-expression of osteoblastogenesis genes, indicating that these compounds can shape the genetic make-up of OS cells and actively suppress their malignant potential, possibly transforming them toward a more normalized and differentiated state. In addition, DNMTi MC3343 improved the accessibility of doxorubicin to DNA, increased stable doxorubicin bonds to DNA, and significantly induced DNA damage and cell death [187]. Overall, these preclinical data indicate that DNMTis may represent a potential therapeutic option for patients with OS who respond poorly to pre-adjuvant chemotherapy. Figure 4 summarizes the involvement of DNA methylation machinery in OS.

### 5.2. Ewing Sarcoma

**Figure 5:**
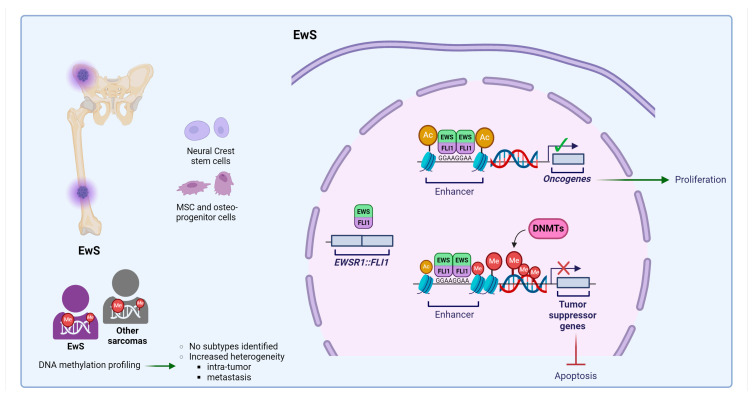
DNA methylation machinery in Ewing sarcomas. Ewing sarcoma (EwS) is a highly malignant neoplasm affecting children and adolescents, and arises in bone and soft tissue from neural crest stem, mesenchymal stem, or osteoprogenitor cells. The tumor is characterized by a reciprocal chromosomal translocation resulting in the formation of an oncogenic chimera, EWS::FLI1. This chimera drives extensive epigenetic reprogramming, affecting key cellular processes. DNA methylation profiling distinguishes EwS tumors with a unique epigenomic signature from other sarcomas. While specific subtypes of EWS are not defined, significant methylation heterogeneity exists within tumors and metastasis. EWS::FLI1 binds to DNA at GGAA sequences, converting them into enhancers with reduced DNA methylation, leading to oncogene activation. Additionally, EWS::FLI1 can recruit repressor complexes in other genome regions, inhibiting tumor suppressor genes via DNA methylation. This results in the induction of proliferation and a reduction in apoptosis. MSCs, mesenchymal stem cells; *EWSR1*, *Ewing sarcoma RNA binding protein 1*; *FLI1*, *Fli-1 Proto-Oncogene ETS transcription factor*; DNMTs, DNA methyltransferases.

EwS represents the second most common primary bone sarcoma in children and adolescents. It is a highly malignant neoplasm that affects both bones and soft tissues, with a high tendency to metastasize. Genetically, EwS is the prototype of sarcomas, which are characterized by a relatively quiet genome [16,190,191], driven by an oncogenic chimera formed as a consequence of a reciprocal chromosomal translocation that fuses a member of the FET family of RNA-binding proteins with a member of the ETS family of transcription factors [192]. The most common translocation, t(11;22)(q24;q12), which is observed in 85–90% of cases, fuses the *Ewing sarcoma RNA binding protein* 1 (*EWSR1)* gene with an ETS family member, most frequently the *Fli-1 Proto-Oncogene* (*FLI1)* gene [192,193], forming the oncogenic driver *EWSR1::FLI1* (or EWS::FLI1). EWS::FLI1 binds to DNA at either ETS-like consensus sites or GGAA microsatellites, which are diverted by the chimera as de novo enhancers [194,195,196]. Through its binding to these sites, EWS::FLI1 reprograms the genetic landscape of EwS, affecting key cellular processes, including cell cycle, apoptosis, angiogenesis, metabolism, and cell migration [192]. Despite the shared molecular basis, the clinical presentation and disease courses of patients with EwS vary considerably. This heterogeneity is not reflected by the genetics of the tumor, but rather depends on epigenetic heterogeneity. A direct link has been identified between the EWS::FLI1 fusion protein and widespread epigenetic reprogramming [196,197,198]. DNA methylation profiling has revealed a characteristic epigenomic signature of EwS that is shared by all EwS tumors, enabling them to be distinguished with remarkable sensitivity and specificity from other tumor types, including Ewing-like sarcomas [70,198,199,200]. However, in contrast to many other cancers, the application of DNA methylation profiling did not result in the identification of discrete subtypes of EwS. Instead, it defined a continuous spectrum between mesenchymal and stem cell signatures, which appeared to be governed by EWS::FLI1 [198]. The most variable DNA methylation levels were observed at the EWS::FLI1 DNA binding site and at repressed genes, which were defined as those that were marked by H3K27ac upon EWS::FLI1 knockdown [197]. In the regions of the genome corresponding to enhancer elements, DNA methylation is significantly reduced, a result consistent with the generation of active enhancers by chromatin decondensation. DNA methylation patterns identify widespread intra-tumor heterogeneity in the same range as that observed for much more genetically heterogeneous cancers and can be linked to genetic and clinical data. Thus, DNA methylation assays identified common distinctive patterns of epigenetic reprogramming that may be used to assess inter- and intra-tumor epigenetic heterogeneity offering new clues for the pathogenesis of EwS and revealing new therapeutic perspectives. As an illustrative example, clusters of cells exhibiting low levels of H3K27ac when EWS::FLI1 is expressed at high levels demonstrated a more pronounced response to treatment with HDAC inhibitors (HDACi) [197]. Over the years, numerous epigenetic inhibitors have been proposed and tested at the preclinical level and/or in phase I/II clinical studies. These include DNMTis, such as decitabine, and the non-nucleoside inhibitor MC3343; HDACis, such as entinostat and belinostat; LSD1 inhibitors, such as INCB059872 and FT-2102 (olutasidenib); and EZH2 inhibitors, such as EPZ6438 (tazemetostat) [201]. Preclinical testing of DNMTis and HDACis has demonstrated that either an agent alone or in combination can inhibit tumor cell growth [202,203,204,205,206,207]. In a study conducted by our group, it was demonstrated that MC3343 DNMTis induced the depletion of DNMT1, resulting in a perturbation of the cell cycle, DNA damage, and cell death [205]. These observations indicate that demethylating drugs may offer potential benefits for the treatment of patients with EwS. Figure 5 outlines the main alterations involving DNA methylation machinery in EwS.

### 5.3. Rhabdomyosarcoma

RMS, the most frequent soft tissue sarcoma found in children, is a high-grade neoplasm composed of cells that have a tendency to differentiate into muscle tissue [208]. The current WHO classification for soft tissue and bone tumors recognizes four distinct subtypes of RMS based on clinicopathological and molecular genetic features: embryonal, alveolar, spindle cell/sclerosing, and pleomorphic subtypes. Embryonal subtypes constitutes 70–80% of all RMS diagnoses and is characterized by aneuploid karyotype that frequently includes chromosome gains of 2, 8, 11, 12, 13, and/or 20, along with losses of chromosomes 10 and 15. Loss of heterozygosity at several loci on chromosome 11 encompasses genes encoding *insulin-like growth factor 2* (*IGF2*) and other growth factors, as well as tumor suppressors like *cyclin dependent kinase inhibitor 1C (CDKN1C)*, which are also frequently observed. In contrast, alveolar RMS constitutes around 20% of all RMS, is more frequently diagnosed in adolescents and young adults, and is genetically characterized by chromosomal translocations involving the *forkhead box transcription factor O1* (*FOXO1*) (13q14.11) that fuses with *Paired Box Transcription Factor 3* (*PAX3*) (2q36.1) in most of the cases (60–70%) or with *Paired Box Transcription Factor 7* (*PAX7*) (1p36.13) (10–20%) [209]. PAX3::FOXO1 binds to specific genomic regions marked by active enhancer chromatin features (H3K27ac), interacts directly or indirectly with chromatin-related proteins (BRD4 and CHD4), and forms complexes with other transcription factors, including N-Myc, MYOD1, and myogenin, generating super-enhancers, associated through 3D looping, with target gene promoters, such as *ALK Receptor Tyrosine Kinase* (*ALK)*, *Fibroblast Growth Factor 4* (*FGFR4*), *MYCN*, *Myogenic Differentiation 1* (*MYOD1*), and *Myogenin* (*MYOG*) [210,211,212]. Genome-wide studies have reported evidence of an association between DNA methylation patterns and histological subtype, as well as clinical outcomes, and revealed novel molecular classes of RMS [70,161,213,214]. 

Analysis of promoter CpG island methylation between RMS subtypes and skeletal muscles revealed RMS-specific hypermethylation in genes associated with tissue development, differentiation, and oncogenesis, such as *DnaJ Heat Shock Protein Family Member A4* (*DNAJA4*), *Hes Family BHLH Transcription Factor 5* (*HES5*), *Iroquois Homeobox 1* (*IRX1*), *Bone Morphogenetic Protein 8* (*BMP8A*), *GATA4*, *GATA6*, *ALX Homeobox 3* (*ALX3*), and *Prolyl 4-Hydroxylase Transmembrane* (*P4HTM*) [214], implicating aberrant DNA methylation in the pathogenesis of RMS. In vitro, DNA methylation changes have been also described in the genes *MYOD* [215], *CDKN1A* [216], *RASSF1* [217], *PAX3* [218], *Junction Plakoglobin* (*JUP*) [219], *FGFR1* [220], *JDP2* [221], *BMP2* [222], and *Caveolin 1* (*CAV1*) [223], compared to normal cells. The role of aberrant DNA methylation in repressing differentiation genes in RMS was indeed demonstrated in early experiments in which the treatment of the RMS cell line RMZ-RC2 with the demethylating agent 5-azacytidine resulted in increased cell differentiation [224]. In contrast, promoter DNA hypomethylation has been found to contribute to the transcriptional activation of a subset of PAX::FOXO1 target genes, indicating a coordinated role in driving target gene expression [225]. Distinct methylation patterns are revealed in alveolar compared to embryonal RMS, with the alveolar subtype being enriched for the DNA hypermethylation of PcG target genes [214]. The DNA methylation pattern was also found to differ in tumors harboring distinct fusion chimeras [225], suggesting that the differences in DNA methylation signatures between RMS subtypes may be useful in defining tumor subtypes, clinical prognosis, and treatment response in RMS tumors.

**Figure 6:**
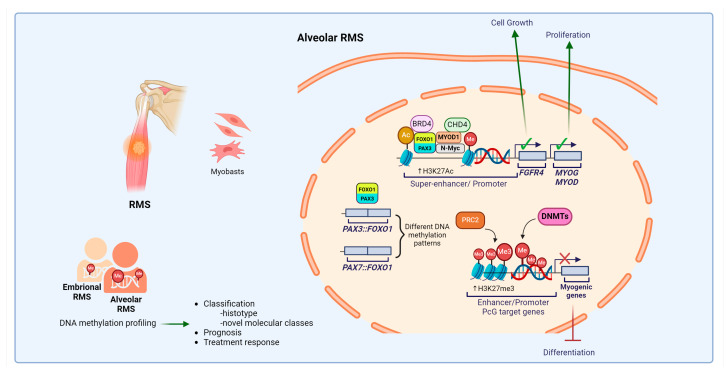
DNA methylation machinery in rhabdomyosarcomas. Rhabdomyosarcoma (RMS), the most common soft tissue sarcoma in children, involves cells differentiating into muscle tissue (e.g., myoblasts). DNA methylation patterns vary by RMS subtype (alveolar and embryonal) and fusion status, affecting classification, prognosis, and treatment response. The alveolar subtype features *PAX3::FOXO1* or *PAX7::FOXO1* translocations. Hypomethylation at PAX3::FOXO1 binding sites on enhancers (H3K27ac) allows the chimera to interact with chromatin proteins (BRD4, CHD4) and transcription factors (N-Myc, MYOD1), forming super-enhancers that in turn activate genes like *FGFR4*, *MYOG*, and *MYOD1*. DNA hypermethylation is enriched in PcG target genes (H3K27me3) inhibiting differentiation. The net result is the promotion of proliferation and cell growth together with the inhibition of cell differentiation. *FOXO1*, *Forkhead Box O1*; *PAX3*, *Paired* Box 3; BRD4, Bromodomain Containing 4; CHD4, Chromodomain helicase DNA binding protein 4; *MYOD1*, *Myogenic differentiation 1*; N-Myc, N-Myc proto-oncogene protein; *FGrR4*, *fibroblast growth factor Receptor 4*; *MYOG*, *Myogenin*; PcGs, Polycomb group proteins; PRC2, Polycomb repressive complex 2; DNMTs, DNA methyltransferases; H3K27me3, histone H3 lysine 27 trimethylation; H3K27Ac, histone H3 lysine 27 acetilation.

From a therapeutic perspective, DNMT3A and DNMT3B levels were found to be significantly upregulated in RMS tumors and cell lines compared to normal skeletal muscle. Accordingly, the depletion of DNMT3B inhibited cell proliferation while promoting myogenic differentiation in different cell lines [187], further supporting the idea that alterations in DNA methylation are critical in the pathogenesis of RMS [226]. The same group further demonstrated that inhibiting DNMT3A and DNMT3B sensitized RMS tumors to radiotherapy. DNMT3A silencing triggered cellular senescence by upregulating p16 and p21 while DNMT3B silencing induced DNA damage and impaired DNA repair machinery, including reductions in ATM, DNA-PKcs, and Rad51 [227]. Exposure of RMS cells to DNMTis or HDACis confirmed pro-differentiating effects by upregulating the expression levels of muscle-specific coding genes and miRNAs [219,228,229]. Additional findings indicated that DNMTis inhibited RMS growth by negatively affecting IGFs, insulin signaling, and the Hippo pathway, which may still be related to differentiation [230,231]. Collectively, these data point to alterations in DNA methylation as a significant contributor to RMS and support a differentiation therapy that envisions the use of DNMTis and other related epigenetic drugs for the treatment of RMS. Figure 6 shows the main role of DNA methylation machinery in RMS.

## 6. DNMT Inhibitors and Epigenetic Reprogramming of Antitumor Immune Responses

Although several epi-drugs have received FDA approval, epigenetic therapy is still in its early stages of development. Preclinical studies have identified a number of promising epigenetic drugs, including inhibitors of DNA methyltransferases (DNMTis), histone deacetylase classes I-II (HDACis), lysine-specific histone demethylase (LSD1i), and histone methyltransferase, such as EZH2i (EPZ6438, Tazemetostat), but their efficient application in clinical settings still needs further research. 

DNMTis can be classified into two main groups: nucleoside analogs and non-nucleoside analogs (Table 1). Nucleoside DNMTis, such as 5-azacitidine and decitabine, have a modified cytosine ring. They can be converted into nucleotides and incorporated into newly synthesized DNA or RNA, resulting in covalent complexes with DNMTs that inhibit DNA methylation [232]. Nucleoside DNMTis have been associated with mutagenic risk, genomic instability, and myelosuppression as side effects [232]. 

Recent research has indeed explored the potential anticancer effects of second-generation nucleoside analog DNMTis, such as guadecitabine, zebularine, TdCyd (tetrahydrouridine-cytidine), and FdCyd (5-fluoro-2′-deoxycytidine). Each analog has specific modifications that affect stability, cellular uptake, and metabolic processing, allowing them to exert their DNMT inhibitory effects for longer periods. They have been studied in different cancer types and contexts, with varying degrees of clinical development and therapeutic potential (Table 1). Guadecitabine (SGI-110) is a dinucleotide of decitabine linked to deoxyguanosine and its structure enhances stability and cellular uptake compared to decitabine alone [233]. It demonstrated efficacy in preclinical models of solid tumors of adult and children, such as RMS, OS, and GBM (Table 1). Zebularine is a cytidine analog that contains a 2(1H)-pyrimidinone ring structure instead of a base [234]. This modification makes it less toxic and more stable than other cytidine analogs and is particularly active in combination treatments resulting in immunomodulatory activities [235,236]. It was effective in preclinical studies (in vitro and/or in vivo) for various pediatric tumors, such as MB, OS, and GBM (Table 1). TdCyd is a combination of tetrahydrouridine (THU), which inhibits cytidine deaminase (CDA), and cytidine. THU prevents the degradation of cytidine analogs by CDA, enhancing their bioavailability and efficacy [237]. It is investigated for its ability to enhance the efficacy of these agents rather than as a standalone therapeutic, and is now in a phase I clinical trial (Table 1). FdCyd is a nucleoside analog of cytidine with a fluorine atom at position 5 of the pyrimidine ring and lacking the 2′-OH group on the sugar moiety, which increases its stability [238]. It has been studied in preclinical models of NB and OS, leading to reduced tumor growth and increased sensitivity to chemotherapy agents (Table 1).

Non-nucleoside DNMTis are able to avoid many side effects. They target DNMTs through various mechanisms, such as direct binding to catalytic sites, the SAM cofactor pocket, or interfering with their binding to DNA [215]. Some of these agents, such as SGI-1027, its quinoline derivatives (MC3343), and GSK3685032 [239,240], have demonstrated sufficient specificity and potency to attract interest for clinical translation. GSK3685032 targets the active site loop of DNMT1 to penetrate hemi-methylated DNA between two CpG base pairs. It exhibits potent antiproliferative activity in various tumors cell lines, with superior hypomethylating activity and lower toxicity compared to other DNMTis [240,241,242]. 

The MC3343 compound was demonstrated to be active in vitro and in vivo, inducing an inhibition of growth and stimulation of cell differentiation in leukemia, MB, OS, and EwS cells (Table 1) [183,187,205]. Notably, this compound appeared to act by inducing the specific depletion of DNMT1, without affecting the general level of DNA methylation [205,240]. In EwS cells, the depletion of DNMT1 by MC3343 resulted in a perturbation of the cell cycle, with an accumulation of cells in the G1 phase, and DNA damage, as evidenced by the induction of γH2AX foci. These effects elicited the activation of p53-dependent signaling and apoptosis in p53 wild-type cells, while in p53 mutated cells, persistent micronuclei and increased DNA instability were observed. Treatment with the MC3343 compound potentiates the efficacy of DNA damaging agents, such as doxorubicin and PARP inhibitors (PARPi) [205], likely because DNMTis increase the amplitude and duration of PARP1 trapping at sites of DNA damage, in this way enhancing its cytotoxic effects [243]. Additionally, DNMTis were demonstrated to induce a BRCAness phenotype, through downregulating the expression of key homologous recombination (HR) and non-homologous end-joining (NHEJ) genes, contributing to synthetic lethality with PARPi in BRCA-proficient cancers [244]. These findings paved the way for a more general application of similar drug combinations in different tumor types. Indeed, in clinical settings, the prevailing approach in phase II trials is to combine epi-drugs with targeted therapy or immune checkpoint blockades in order to enhance the general therapeutic efficacy. Advances in the use of epigenetic and immunotherapeutics together with recruiting clinical studies in pediatric solid tumors have been recently reviewed [201,245,246].

**Table 1:** DNMT inhibitors in pediatric cancers. ↑, Induction; ↓, reduction; n.a., not applicable; MB, medulloblastoma; NB, neuroblastoma; ETMRs, embryonal tumor with multilayered rosettes; OS, osteosarcoma; EwS, Ewing sarcoma; RMS, rhabdomyosarcoma; GBM, glioblastoma; GIST, gastrointestinal stromal tumor; MPNST, malignant peripheral nerve sheath tumor; TMZ, Temozolomide; PARPi, PARP inhibitor.

Group	Mechanism of Action	Drug	Target(s)	Tumor	Results		References
					Preclinical	Clinical	
Nucleoside analogs	Cytidine replacement in DNA, DNMT1 blocking, DNMT degradation, and DNA demethylation	5-azacitidine (5-AZA)	DNMT1	Glioma	↓ DNA methylation ↓ cell proliferation ↓ tumor growth ↑ cell differentiation ↑ TMZ efficacy	n.a.	[94,97,98]
				MB	↑ cell death ↑ HDACi efficacy ↓ tumor growth	Phase I NCT06466798	[118,122]
				ETMR	↓ cell proliferation	n.a.	[126]
				OS	↓ cell proliferation ↑ cell death ↑ cell differentiation ↑ HDACi efficacy overcome chemoresistance	n.a.	[188,189]
				RMS	↓ cell proliferation ↑ cell differentiation	n.a.	[224,230]
		5-aza-2′-deoxycytidine (decitabine)	DNMT1	Glioma	↓ DNA methylation ↓ tumor growth ↑ cell differentiation ↓ migration ↓ invasion	Phases I/II NCT02332889	[93,95,96,99,100]
				MB	↓ cell proliferation ↑ cell death ↓ tumor growth ↑ cell differentiation ↑ HDACi efficacy	Phases I/II NCT02332889	[119,120,121,217]
				ETMR	↓ cell proliferation ↑ cell death ↑ Topotecan efficacy	Phases I/II NCT02332889	[128]
				NB	↓ DNA methylation ↑ cell death ↓ cell proliferation ↑ cell differentiation ↑ cisplatin, doxorubicin, and etoposide efficacy ↑ Retinoid acid efficacy	Phase I NCT00075634 NCT01241162	[121,153,154,155,156,217,247,248]
				OS	↓ DNA methylation ↓ cell proliferation ↑ cell death ↓ tumor growth ↑ immune response ↓ metastases	Phase I NCT01241162	[176,181,184,185,186,187,217,247,249,250]
				EwS	↓ DNA methylation ↓ cell proliferation ↑ cell death	Phase I NCT01241162	[202,203,204,205,206,207,217,247]
				RMS	↓ DNA methylation ↓ cell proliferation ↓ tumor growth ↑ HDACi efficacy ↑ IFNγ response	Phase I NCT01241162	[120,216,217,219,222,223,247]
		Guadecitabine (SGI-110)	DNMT1	GBM	↓ DNA methylation ↓ tumor growth ↓ invasion ↑ immune response ↑ IFN I/II/III responses	n.a.	[251]
				RMS	↓ DNA methylation ↓ cell proliferation ↑ cell death ↑ cell differentiation ↓ tumor growth	n.a.	[231,252]
				OS	↑ immune response	n.a.	[253]
				GIST, adult sarcoma	n.a.	Phase II NCT03165721 NCT04340843	[254]
		Zebularine	DNMT1	GBM	↓ DNA methylation ↓ cell proliferation	n.a.	[255]
				MB	↓ cell proliferation ↑ cell death ↑ vincristine efficacy ↑ TLRs pathway	n.a.	[256]
				OS	↓ DNA methylation ↓ cell proliferation ↑ cell death	n.a.	[257,258]
		4′-thio-2′-deoxycytidine (TdCyd)	DNMT1	adult solid tumors	↓ DNA methylation ↓ cell proliferation ↓ tumor growth	Phase I NCT02423057	[237,259]
		5-fluoro-2′-deoxycytidine (FdCyd)	DNMT1	MB	↓ cell proliferation no response in vivo	n.a.	[260]
				OS	↓ cell proliferation ↑ DNA damage response	n.a.	[261]
				adult solid tumors	↓ cell proliferation	Phase I NCT00378807 NCT00978250	[262,263]
Non-nucleoside analogs	Blocking of DNMT catalytic pocket/allosteric sites/DNA interaction	GSK3685032	DNMT1	MPNST	↓ cell proliferation ↑ cell death ↑ viral mimicry response ↑ immune response	n.a.	[242]
		SGI-1027 and derivatives (MC3343)	DNMT1, DNMT3A/B	MB	↓ cell proliferation ↑ cell differentiation	n.a.	[183]
				OS	↓ cell proliferation ↑ cell death ↑ cell differentiation ↑ doxorubicin and cisplatin efficacy	n.a.	[187]
				EwS	↓ cell proliferation ↑ cell death ↑ DNA damage response ↑ doxorubicin, PARPi efficacy	n.a.	[205]

A growing body of evidence indicates that epigenetic drugs also possess the ability to modulate antitumor immune responses. This can be achieved either by influencing the immunogenicity of tumor cells or by directly affecting immune cells that are functionally involved in antitumor responses [264]. It becomes evident that an understanding of the role of epigenetic regulators in cancer immunity is of paramount importance in order to fully harness the potential of epigenetic drugs. Despite the direct antiproliferative, pro-differentiative antitumor effects elicited by DNMTis, responses in the clinic were frequently delayed and low-dose treatment regimens were found to be the most effective, supporting the idea that clinically relevant antitumor activities of DNMTis are beyond tumor cell intrinsic effects. Indeed, gene expression profiling of primary tumors treated with DNMTis reveals an enrichment for immune-related pathways [265,266]. Additionally, hypomethylation can increase the expression of genes associated with antigen presentation and immune co-stimulatory molecules, such as *CD80*, *CD86*, and *CD40*, thus enhancing tumor cell immunogenicity [264]. Conversely, aberrant DNA methylation in tumor cells correlates with reduced immune infiltration and poorer prognosis in several tumor types, including some “big killers”, such as breast and colon cancers [267,268]. In glioma, the presence of IDH mutation was found to correlate with reduced immune cell infiltration [269]. Dysregulated DNA methylation negatively influences STAT1 expression. As a result, interferon-gamma (IFNγ) signaling is impaired, leading to the reduced expression of major histocompatibility complex class I molecules (MHC-Is) and type 1 T-helper (T_H_1) chemokines CXCL9/CXCL10 [270,271]. Furthermore, the expression of the programmed death-ligand 1 (PD-L1) checkpoint is regulated through the DNA methylation of AP-2α, which has a low expression in high-grade gliomas [272]. Overexpressed AP-2α in gliomas not only inhibits the transcription of PD-L1, but enhances the endocytosis and degradation of PD-L1 proteins in tumor cells and increases CD8+ T cell-mediated proliferation, effector cytokine secretion, and cytotoxicity in vitro, supporting a combination of demethylating drugs with anti-PD-1 immunotherapy to efficiently suppress the progression of gliomas [272].

DNA methylation also plays a role in repressing the transcription of transposable elements, including endogenous retroviruses (ERVs), within the host genome [67]. Hypomethylating agents can stimulate the re-expression of ERVs within tumor cells, which triggers innate antiviral-like responses, a mechanism known as viral mimicry [273,274]. The bidirectional transcription of ERVs generates double-stranded RNA (dsRNA) that is detected by various pattern recognition receptors, including Toll-like receptors (TLRs) and cytosolic receptors, such as MDA5, RIG-I, and cGAS-STING pathways [273]. The activation of dsRNA-sensing pathways results in the induction of type I interferons (IFNα and IFNβ) via pro-inflammatory transcription factors, such as IRF7 and NF-κB [273]. Autocrine and paracrine signaling of IFNα/β stimulate the release of pro-inflammatory cytokines and chemokines into the tumor microenvironment (TME). The net result of this response is a reduction in the fitness of cancer cells and an increase in the attraction of cytotoxic T lymphocytes (CTLs) to the TME [251,264,273,274,275]. A further advantage of the process is to induce a bystander effect against tumor cells that were not directly affected by the activity of the DNMTis because they were not in the S phase at the time of exposure, further potentiating immune checkpoint therapy. 

We must consider that cytosine methylation plays a pivotal role in regulating a large number ERVs. Consequently, it has been demonstrated that DNMTi exposure can rapidly and effectively reactivate these elements and induce the production of neoantigens by ERV-derived transcripts, thereby synergizing with immune therapies [276]. Furthermore, DNMTi treatment was found to result in the expression of thousands of non-annotated transcription start sites, which can lead to the production of truncated or chimeric proteins. These proteins may act as neo-antigens, which could be presented on MHC-I for recognition by CD8+ T cells. The results of these studies indicate that the reactivation of human ERVs via epigenetic therapy has the potential to modulate the immune TME and enhance the response to immunotherapy. This is particularly interesting for the ‘cold’ microenvironment of pediatric tumors. In gliomas, the cold TME can be rescued by DNMTis, which also induce the reactivation of the *Stimulator of Interferon Genes* (*STING*) [277,278]. In MB, several pathways related to immune response were found to be modulated by DNMTis, including the toll-like receptor pathway [256]. In preclinical and clinical studies of relapsed high-risk or therapy-refractory NB, OS, EwS, or RMS, DNMTis have been shown to upregulate tumor-specific antigen expression, increase susceptibility to T-cell-mediated killing, and enhance the efficacy of combined treatment with a dendritic cell vaccine [247,249,250,279]. In a mouse model of EwS, abemaciclib, a CDK4/6 inhibitor, was shown to reduce DNMT1 expression, which in turn stimulated an inflammatory immune response through increased cytokine secretion, antigen presentation, and upregulation of the interferon pathway [280]. In high-grade MPNST, an aggressive sarcoma with poor prognosis and no effective targeted therapy, the targeting of DNMT1 with decitabine or DNMT1-selective catalytic inhibitor GSK3685032 caused the transcriptional activation of ERV retrotransposons and subsequent viral mimicry-mediated cell death [242]. The effects of DNMTi-induced viral mimicry were amplified by the presence of inactivating mutations in the PRC2 proteins, a condition present in around 80% of MPNSTs [281], priming for selective tumor cell death while sparing normal cells. Functional inactivation of PRC2 via oncohistone mutation (H3 K27M) was also observed in pediatric gliomas [282], suggesting that it is possible to set up a tumor context-specific liability for therapeutic intervention via DNMT1 inhibitors in selective cancer types.

Nevertheless, it is not clear whether all immunological changes induced by hypomethylating agents are beneficial in promoting antitumor responses. It is also uncertain whether different classes of repeat elements are differently sensed by cells, leading to distinct downstream biological responses [67].

In addition to cancer cells, infiltrating immune cells also exhibit epigenetic abnormalities. Thus, DNMTis have the potential to enhance antitumor immune responses, acting on both cancer cells and immune cells. For instance, NK cells treated with decitabine demonstrate enhanced effector functions [283], thereby enhancing antitumor immunity. The exhausted phenotype of cytotoxic T lymphocytes (CTLs) is characterized by aberrant DNA methylation at genes associated with T-cell effector functions. The use of DNMTis can prevent the onset of exhaustion and reprogram exhausted CTLs into effector phenotypes [284]. In Treg cells, EZH2i or DNMTis can reduce the expression of immunosuppressive genes like *Cytotoxic T-Lymphocyte Associated Protein 4* (*CTLA4*), potentially enhancing antigen presenting cell co-stimulation of CD8+ T cells [264]. Additionally, monocytes, dendritic cells, and myeloid-derived suppressor cells (MDSCs) exhibit varying DNA methylation patterns around genes linked to their suppressive abilities. Inhibiting DNMTs can reduce MDSC frequency within tumors, thereby potentially improving antitumor immune responses [285]. 

## 7. Conclusions

The findings reviewed here indicate that DNA methylation profiles play a significant role in the refinement of pediatric tumor diagnosis, with more accurate subtype distinctions and an improved evaluation of patient risks. Epigenetic therapy with DNMTis may exert antiproliferative and pro-apoptotic effects, while promoting cell differentiation. Therefore, these epi-drugs have the potential of redirecting cancer cells toward a more normalized state. Moreover, recent discoveries have highlighted the potential clinical applications of DNMTis in combination with immunotherapy approaches. The combination of DNMT inhibition and immunotherapies to elicit more potent antitumor immune responses represents an important novel therapeutic avenue for tumors that still have unmet needs. Nevertheless, further research is necessary to: i. improve our understanding of the complexity of DNA methylation regulation and the multitude of molecular processes involved; ii. determine the impacts of the dosing and scheduling of demethylating drugs in the clinical setting and in relation to immune responses; and iii. set-up tumor context-specific conditions for therapeutic intervention via DNMT1 inhibitors to avoid unwanted toxicity. The function of DNA methylation is cell context-dependent both in terms of genomic features and the broader interactions with tumor microenvironment cellular components. It is thus essential to identify appropriate experimental preclinical models that not only recapitulate the cellular aspects of the targeted tumor, but also the specific immune context in order to ensure an accurate representation of the therapeutic effects. This aspect is particularly relevant for rare, pediatric tumors considering that providing new therapy to children with cancer has additional challenges compared with adult malignancies. 

## Data Availability

No new data were created and analyzed in this study.
